# Does the Surgical Approach Influence the Canal Fill of the Proximal Femur for Hip Arthroplasty?

**DOI:** 10.1016/j.artd.2021.05.011

**Published:** 2021-08-07

**Authors:** Lucas Mattesi, Adrien Cheyrou-Lagrèze, Guillaume-Anthony Odri, Antoine Duhil, Laure Flurin, Mathieu Severyns

**Affiliations:** a Orthopaedic and Traumatologic Department, CHU Martinique, Fort-de-France, France; b Orthopaedic Department, CHU Lariboisière, Paris, France; c Divisions of Clinical Microbiology and Infectious Diseases, Mayo Clinic, Rochester, MN, USA

**Keywords:** Hip arthroplasty, Canal fill ratio, Canal calcar index, Dorr’s classification

## Abstract

**Background:**

Choosing the right size of the stem is crucial for uncemented hip arthroplasty. Undersizing can lead to early loosening, peri-prosthetic fracture due to femoral implant insertion, and/or osteointegration failure. The main objective of this study was to find a correlation between the surgical approach and the intramedullary prosthetic canal fill ratio (CFR) of the uncemented femoral implant. The hypothesis of this work was that the surgical approach does not influence the stem sizing during hip arthroplasty.

**Methods:**

In this consecutive series, we analyzed the radiological images of 183 patients who underwent primary hip arthroplasty with 4 different surgical approaches. Dimensions of the implant were evaluated by radiographic measurement of the CFR. In order to assess the shape of the femur, we measured the canal flare index on the preoperative radiographs, and the canal calcar ratio was also measured to establish the shape of the femur according to Dorr's classification.

**Results:**

No significant difference was found between the surgical approach and the CFR measured at 4 different levels (CFR 1, 2, 3, and 4) on the postoperative radiograph. When the shape of the femur was assessed by canal flare index, there was no significant difference in implant, whether the femur had a stovepipe canal shape or a champagne-fluted canal shape.

**Conclusion:**

This study showed that the surgical approach in hip arthroplasty does not influence the canal fill. Therefore, the surgical approach does not factor in undersizing the femoral implant. Despite some difficulties in the exposure of the medullary shaft described by some authors, the anterior approach is not a risk factor for undersizing an anatomical femoral stem.

**Level of evidence:**

4

## Introduction

Hip arthroplasty offers excellent functional results and long-term survival rates [1,2]. Uncemented hip implants are often preferred thanks to good stability, as well as excellent long-term osteointegration [3]. Choosing the right size is crucial for uncemented prosthesis. Too small, it can lead to early loosening, peri-prosthetic fracture due to femoral implant insertion, and osteointegration failure [[4], [5], [6], [7]]. On the contrary, a good stem sizing allows good stress distribution on the proximal femur [8] and good biological anchorage [9,10]. There are significant anatomical variations in the shape of the proximal femur. This morphological variability is a major source of error in the choice of implant size which later will lead to early revision [[11], [12], [13]]. The choice of approach also appears to influence the canal fill. Some authors found that an anterior approach often leads to femoral implant undersizing, which could also be explained by a longer learning curve for this approach [14]. Given the risk of fracture and the difficulties of osteosynthesis, many surgeons find it better to use successive femoral rasp for anterior or anterolateral approaches. Overall, undersizing of the intramedullary shaft may therefore be made difficult by the bone structure, the femoral offset, the intramedullary caliber, and the surgical approach.

The main objective of this study was to find a correlation between the surgical approach and the intramedullary prosthetic canal fill ratio (CFR) of the uncemented femoral implant. The hypothesis of this work was that the surgical approach does not influence the stem sizing during hip arthroplasty. To complete this analysis of the canal fill, we also evaluated the morphology of the recipient femurs.

## Material and methods

### Population

We conducted a retrospective study in our university hospital. We analyzed the radiological images of 183 patients who underwent primary hip arthroplasty (total or hemiarthroplasty) between January 2016 and December 2018.

The inclusion criteria were (1) patient aged >18 years, (2) who had undergone primary hip arthroplasty, (3) with an uncemented Hip'n'go femoral stem (FH Orthopedics, Heimsbrunn, France), and (4) who had a preoperative and a postoperative frontal Radiograph of the hip.

Patients were then categorized into 4 groups according to the surgical approach performed for the hip arthroplasty. Group 1 consisted of patients undergoing Hueter anterior approach without an orthopedic table (n = 40), group 2 of patients undergoing Rottinger anterolateral approach (n = 53), group 3 of patients undergoing modified Hardinge lateral approach (n = 50), and group 4 of patients undergoing Moore posterolateral approach (n = 40). A total of 4 surgeons operated on these patients, each surgeon making only his own approach. No intraoperative Radiograph control was performed during the surgical procedure.

The femoral stem used was a cementless wedge taper for more of a fit and fill–type stem with a frontal curve and straight sagittal design. It should be noted that the femoral preparation technique for this anatomic stem consists in reaming and broaching.

### Evaluation criteria

**Figure 1:**
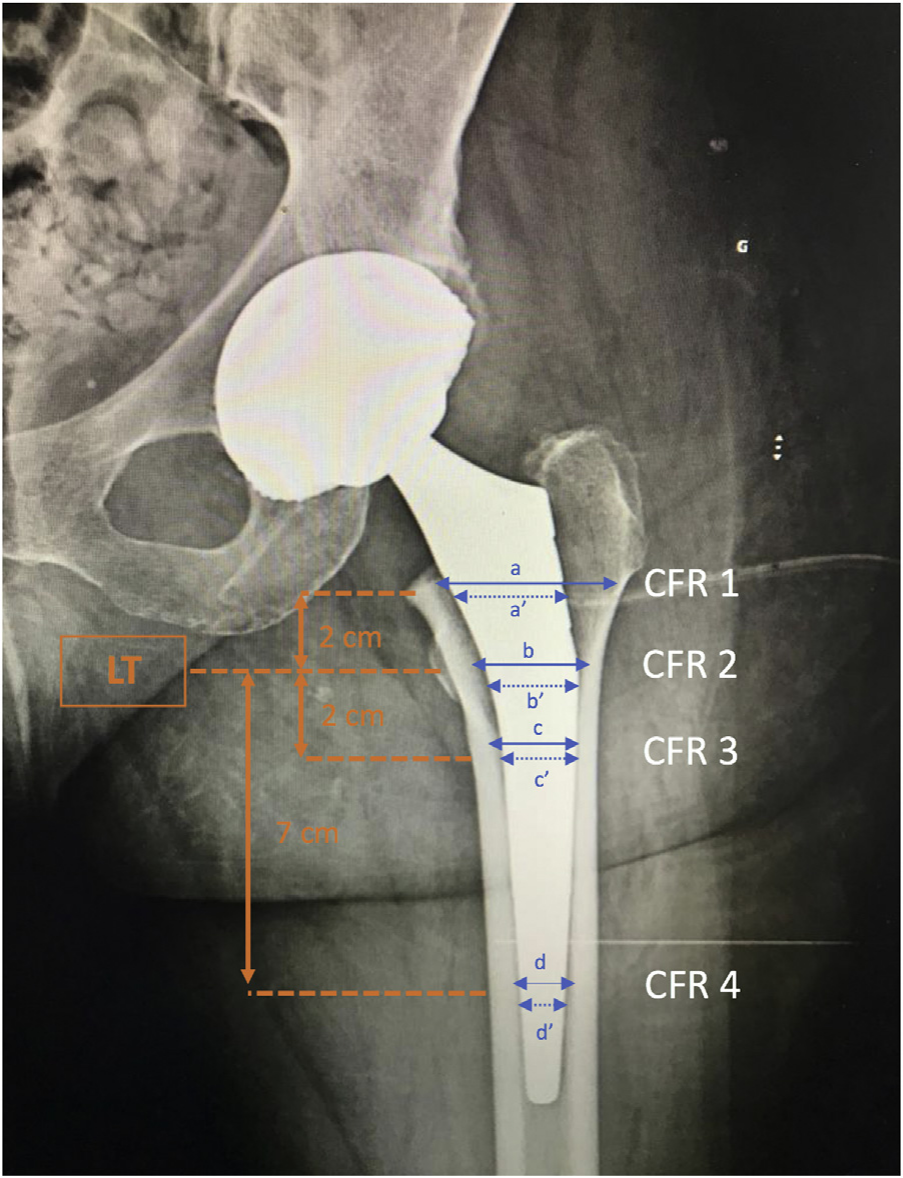
Dimensions of the implant were evaluated by radiographic measurement of the canal fill ratio (CFR) at 4 different levels on the postoperative radiograph: at the level of the lesser trochanter, 2 cm above and below the lesser trochanter and 7 cm below the lesser trochanter.

The dimension of the implant was evaluated by radiographic measurement of the CFR [15] at 4 different levels on the postoperative radiograph: at the level of the lesser trochanter, 2 cm above and below the lesser trochanter and 7 cm below the lesser trochanter (Fig. 1).

**Figure 2:**
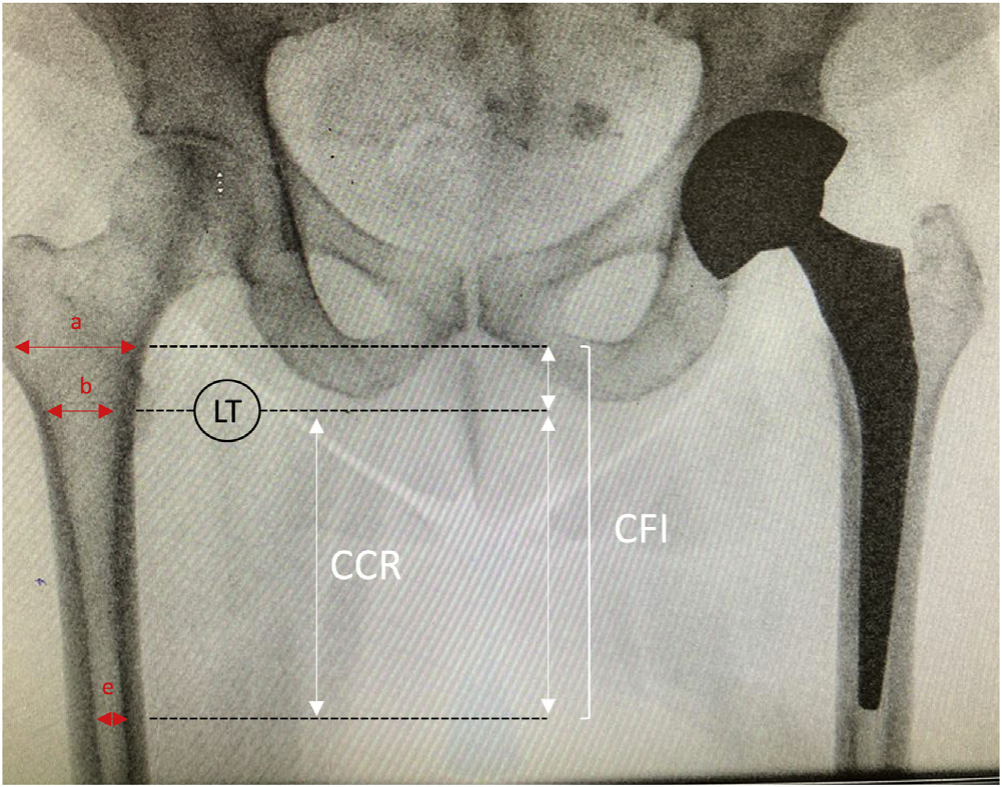
The canal flare index (CFI) was defined by the width of the medullary canal 2 cm above the lesser trochanter, divided by the width of the canal 10 cm below the lesser trochanter. Canal calcar ratio (CCR) was defined as the width of the medullary canal 10 cm below the lesser trochanter divided by the width of the canal at the lesser trochanter.

**Figure 3:**
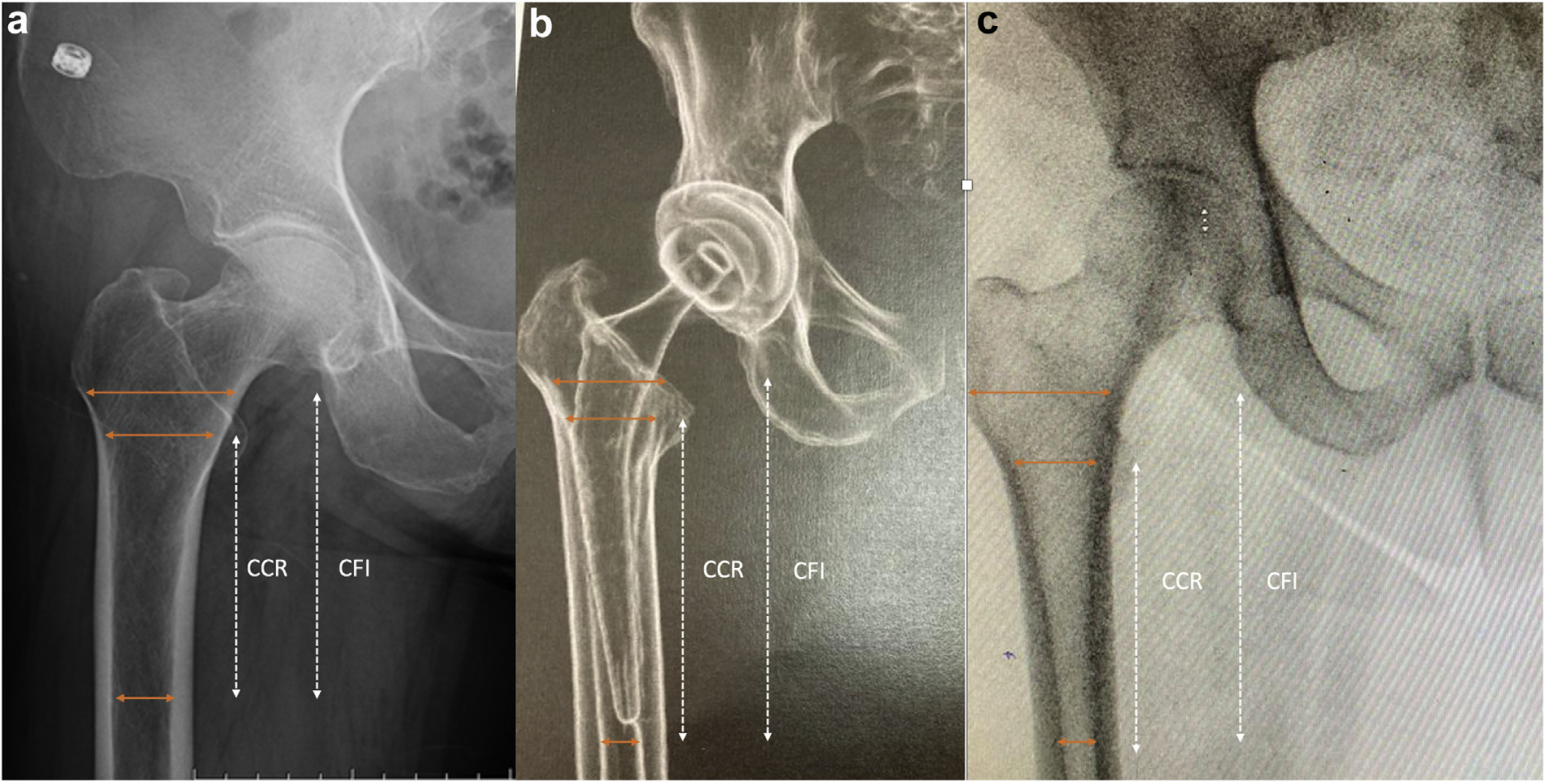
Radiograph control of a CFI < 3.0 (a, stovepipe-canal shape femur), (b) a CFI between 3.0 and 4.7 was normal canal shape, and (c) a CFI > 4.7 was considered a champagne-fluted canal shape.

In order to assess the shape of the femur, we measured the canal flare index (CFI) [16] on the preoperative radiographs (Fig. 2). The CFI was defined by the width of the medullary canal 2 cm above the lesser trochanter, divided by the width of the canal 10 cm below the lesser trochanter. According to the criteria of Noble et al. [16], a CFI < 3.0 was considered a stovepipe-shape femur, a CFI between 3.0 and 4.7 was considered an intermediate form, and a CFI > 4.7 was considered a champagne-fluted canal shape (Fig. 3). The canal calcar ratio (CCR) [17] was also measured to establish the shape of the femur according to Dorr's classification [18]. CCR was defined as the width of the medullary canal 10 cm below the lesser trochanter divided by the width of the canal at the lesser trochanter. A CCR < 50% was classified as Dorr A femur, a CCR between 50 and 75% was classified as Dorr B femur, and a CCR > 75% was classified as Dorr C. Radiographic analysis was performed blindly and independently by 2 senior surgeons.

### Statistical analysis

Data were collected in an Excel spreadsheet (Microsoft, Richmond, WA) and analyzed with JMP 10.0 software (SAS Inc., Cary, NC) via a protocol validated by the institutional review board, which is a part of our institution's research department (institutional review board reference number: 2020/083). A Shapiro-Wilk test was performed to test the normal distribution of quantitative variables. A post-hoc test was performed to compare the mean of multiple quantitative variables with normal distribution. The significance threshold was then *P* < .05 for all tests. Interobserver correlation of the radiographic measurements was measured by the Kappa correlation coefficient, and interobserver agreement was given in percent.

## Results

A total of 183 patients were included; 110 were women and 73 were men. The average age was 74.4 ± 13.2 years. One hundred eight underwent total hip arthroplasty, and 75 underwent hemiarthroplasty. Between total arthroplasty and hemiarthroplasty, we observed a similar distribution within different surgical approaches (*P* = 0.15). All patient characteristics are detailed in Table 1.

**Table 1:** Patient characteristics.

Surgical approach	Hueter	Rottinger	Hardinge	Posterior	*P* value
N	40	53	50	40	
Age	73.0 (±11.9)	78.2 (±14.9)	73.16 (±14.8)	72.2 (±12.3)	.11
Gender (M/F)	19/21	20/33	18/32	16/24	.059
Side (R/L)	18/22	24/29	20/30	24/16	.218
HA/THA	17/23	35/18	12/38	11/29	<.0001
CFI					
Stovepipe canal shape	22	39	30	26	NS
Normal canal shape	15	12	18	13	
Champagne-fluted canal shape	3	2	2	1	
Dorr classification (CCR)					
Dorr A	23	15	21	20	NS
Dorr B	14	34	26	17	
Dorr C	3	4	3	3	

HA, hemiarthroplasty; THA, total hip arthroplasty.

### Stem sizing and surgical approach

No significant difference was found between the surgical approach and the CFR measured at 4 different levels (CFR 1, 2, 3, and 4) on the postoperative radiograph. Therefore, implant size was not dependent on the surgical approach (Table 2).

**Table 2:** Relation between canal fill ratio (CFR) and surgical approach.

Surgical approach	Hardinge	Hueter	Posterior	Rottinger	*P*
N	50	40	40	53	
CFR > 2 cm LT	0.65 ± 0.11	0.62 ± 0.09	0.67 ± 0.17	0.63 ± 0.11	.23
CFR LT	0.70 ± 0.09	0.72 ± 0.09	0.72 ± 0.08	0.72 ± 0.12	.71
CFR < 2 cm LT	0.73 ± 0.10	0.74 ± 0.09	0.74 ± 0.11	0.77 ± 0.13	.40
CFR < 7 cm LT	0.74 ± 0.11	0.80 ± 0.11	0.77 ± 0.14	0.79 ± 0.10	.11
Canal calcar ratio (CCR)	0.53 ± 0.11	0.50 ± 0.10	0.53 ± 0.12	0.54 ± 0.13	.40

LT, lesser trochanter.

### Stem sizing, surgical approach, and femoral shape

When the shape of the femur was assessed by CFI, there was no significant difference in implant, whether the femur had a stovepipe canal shape or a champagne-fluted canal shape.

In the normal canal shape group, there was a significant difference in the dimensions of the lesser trochanter. After the post-hoc analysis, the Rottinger group had more prosthetic space in the lesser trochanter than the Hueter, Hardinge, and Moore groups. (*P* < .091).

When comparing the shape of the femur according to Dorr's classification using CRC measurement, there was no significant difference in canal fill between the 4 surgical approaches.

### Relation between canal fill and canal shape

After a comparison between CFI and CFR, stovepipe canal shape had a low distal canal fill requirement, while champagne-fluted canal shape femurs had a high distal canal fill requirement (Table 3).

**Table 3:** Relation between canal fill ratio (CFR) and canal shape.

Canal shape	Stovepipe canal shape (1)	Normal canal shape (2)	Champagne-fluted canal shape (3)	*P* value	Post-hoc test
CFR > 2 cm LT	0.68 ± 0.13	0.58 ± 0.06	0.57 ± 0.04	<.0001	1 > 2 and 3
CFR LT	0.72 ± 0.11	0.71 ± 0.09	0.64 ± 0.10	.13	NS
CFR < 2 cm LT	0.73 ± 0.12	0.77 ± 0.10	0.70 ± 0.10	.09	NS
CFR < 7 cm LT	0.74 ± 0.11	0.84 ± 0.10	0.83 ± 0.13	<.0001	1 < 2 and 3

LT, lesser trochanter.

Similarly, when considering CFR, recipient femurs classified as Dorr A had a better CFR distally than proximally. The reverse was verified with greater canal fill in the proximal than in the distal region in the case of Dorr C femurs (Table 4).

**Table 4:** Relation between canal fill (CFR) and Dorr’s classification.

Dorr’s classification	Dorr A (1)	Dorr B (2)	Dorr C (3)	*P* value	Post-hoc test
CFR > 2 cm LT	0.61 ± 0.12	0.66 ± 0.11	0.71 ± 0.14	.0026	1 < 2 and 3
CFR LT	0.68 ± 0.09	0.74 ± 0.09	0.80 ± 0.14	<.0001	1 < 2 < 3
CFR < 2 cm LT	0.77 ± 0.12	0.74 ± 0.10	0.72 ± 0.13	.15	NS
CFR < 7 cm LT	0.84 ± 0.10	0.75 ± 0.10	0.63 ± 0.12	<.0001	1 > 2 > 3

LT, lesser trochanter.

For all radiographic measurements, interobserver agreement was 98% with a Kappa correlation coefficient of 0.96 (0.88-1).

## Discussion

This study showed that the surgical approach in hip arthroplasty does not influence the canal fill. Therefore, the surgical approach does not factor in undersizing the femoral implant. Undersizing of the femoral implant is a risk factor for early loosening. As shown by Fottner et al. [19], undersizing of the stem leads to increased micromovements and increased shielding stress. Angerame et al. showed that revision surgery for early femoral implant loosening occurs more frequently in the anterior approach and that loosening occurs most frequently in Dorr A femurs when using an anterior tract [20]. In this study, we showed that, regardless of the Dorr femur shape, the approach does not affect the sizing of the implant. This early loosening in Dorr A femurs when using an anterior approach is more likely to be related to a metaphyseal crowding defect than to the approach itself. Indeed, as Park et al. showed, the survival rate of uncemented stems is lower in Dorr A femurs than in B femurs [21]. It appears that the determining factor in the survival of the femoral implant is primarily metaphyseal canal fill, which is mainly related to the design of the femoral implant. In order to make the comparison of the prosthetic dimensions feasible and reliable, we used a single femoral implant design, an anatomical stem. However, Janssen et al. [22] showed in their study that early loosening of the so-called straight femoral implants is more frequent in the anterior and anterolateral surgical approaches than in the posterior approach, but the study showed no difference with anatomical implants. This could be explained by the fact that in the anterior and anterolateral tracts, the femoral exposure is more complex, and therefore, the metaphyseal preparation would be not as good, which could lead to malposition and undersizing of the straight implant. However, this was not confirmed in our study with an anatomical implant. It is important to specify that an impaction broach stem can appear to be “undersized” based on radiographs but with a high intraoperative stability.

Another limitation is that we conducted a retrospective study of radiographic measurements, without patient follow-up and therefore without evaluation of osteointegration and loosening. Graw et al. showed that minimally invasive pathways are a risk factor for early failure of osseointegration and early loosening [23].

It is important to point out that some authors have found that the choice of surgical approach influences the development of periprosthetic osteolysis due to proximal femur stress shielding variation [24,25]. One of the reasons given for this variation is the tissue damage caused during the surgical approach which could modify the stress shielding distribution in the proximal femur. Muscle balance around the femur could change the distribution of applied forces and thus the stress shielding in the end. It is also for this reason that the onset of early loosenings in the anterior and minimally invasive tracts is unclear. It is often attributed to the difficulty of exposure which leads to undersizing or malpositioning the implant. However, our work confirms that, despite some difficulties in the exposure of the medullary shaft described by some authors, the anterior approach is not a risk factor for undersizing an anatomical femoral implant.

## Conclusions

The surgical approach in hip arthroplasties does not influence the implant dimensions and is therefore not a factor for undersizing of the femoral stem. These data are based on a continuous series, that is, the first study with a comparison between 4 different surgical approaches. Although femoral canal fill is closely related to the morphology of the recipient’s femur, no relationship was found between the surgical approach, the shape of the femur, or stem sizing.

## Funding

There is no funding source.

## Conflicts of interest

The authors declare that they have no known competing financial interests or personal relationships that could have appeared to influence the work reported in this article.

## Appendix A Supplementary data

Conflict of Interest Statement for Cheyrou-Lagrèze

Conflict of Interest Statement for Duhil

Conflict of Interest Statement for Flurin

Conflict of Interest Statement for Mattesi

Conflict of Interest Statement for Odri

Conflict of Interest Statement for Severyns
